# Study of Allergic Rhinitis in Childhood

**DOI:** 10.1155/2011/487532

**Published:** 2011-06-28

**Authors:** Dimitrios G. Balatsouras, George Koukoutsis, Panayotis Ganelis, Alexandros Fassolis, George S. Korres, Antonis Kaberos

**Affiliations:** ^1^ENT Department, Tzanion General Hospital of Piraeus, Afentouli 1 & Zanni, 18536 Piraeus, Greece; ^2^ENT Department, Atticon University Hospital of Athens, 1 Rimini Str., Haidari, 12462 Athens, Greece

## Abstract

Allergic rhinitis is common among children and quite often represents a stage of the atopic march. Although sensitization to food and airborne allergens may appear in infancy and early childhood, symptoms of the disease are usually present after age 3. The aim of this study was to determine the most frequent food and indoor and outdoor respiratory allergens involved in allergic rhinitis in children in the region of Piraeus. The study was performed in the outpatient clinic of otolaryngologic allergy of a general hospital. Fifty children (ranged 6–14 ) with symptoms of allergic rhinitis and positive radioallergosorbent test (RAST) for IgE antibodies or skin prick tests were included in the study. Thirty six (72%) of the subjects of the study had intermittent allergic rhinitis. The most common aeroallergens determined were grass pollens and Parietaria, whereas egg and milk were the food allergens identified. The detection of indoor and outdoor allergens in the region of Piraeus, based on skin prick tests and RAST tests, showed high incidence of grasses and food allergens, which is similar to other Mediterranean countries.

## 1. Introduction

Allergic rhinitis is one of the most common disorders, which affects 5–40% of population, according to various reports [1, 2]. It may be classified as persistent and intermittent allergic rhinitis, depending on the frequency of symptoms. It presents a high morbidity because it affects social life, professional activities, and, especially in children, school performance [3]. 

Allergic rhinitis is common among children and quite often represents a stage of the atopic march [4]. Although sensitization to food and airborne allergens may appear in infancy and early childhood, symptoms of the disease are usually present after age 3. The aim of this study was to determine the most frequent respiratory and food allergens as a cause of allergic rhinitis in children in the region of Piraeus.

## 2. Materials and Methods

We examined 50 children who presented with allergic rhinitis in the outpatient clinic of otolaryngologic allergy, belonging to the ENT department of our hospital. The age of the patients ranged from 6 to 14 years (mean 10.7 ± 2.1), and they were all living in the region of Piraeus. Diagnosis was on the basis of a history of allergic rhinitis, either seasonal or perennial, on the findings of clinical examination and on the presence of positive radioallergosorbent (RAST) test for IgE antibodies (RAST-CAP-FEIA, Pharmacia, Uppsala, Sweden). All children were tested in a series of allergens, including grasses, cereales, parietaria, urtica, tree allergens (*Olea europea*, *Cypressus sempervirens*, *Pinus pinea*, and *Populus alba*), dust mites, animal dander and food allergens. The RAST results were classified, as Class 1 (low level of specific IgE), Class 2 (moderate level), Class 3 (high level), and Class 4 (very high level) [5]. In a group of 12 older children, skin-prick tests were also performed. Details of this procedure are reported elsewhere [6]. The skin prick tests were considered positive if the mean wheal diameter was 3 mm or larger.

## 3. Results and Discussion

The main clinical and demographic features of our patients are shown in Table 1. Twenty-nine patients were females and 21 males. Severity of rhinitis was mild in 19 patients (38%) and moderate/severe in the remaining 31 (62%), according to the criteria of ARIA [7]. Thirty-seven (74%) of the children suffered from other allergic diseases as well, including childhood asthma, allergic conjunctivitis, and atopic dermatitis.

Thirty six (72%) of the subjects of the study had intermittent allergic rhinitis, owed to pollen aeroallergens, with a mean of 2 allergens per patient (Tables 2 and 3). Nine patients (18%) were sensitized to one allergen, 17 patients (34%) were sensitized to two and 10 patients (20%) to three allergens. The remaining 14 (28%) children suffered from persistent rhinitis, owed mainly to nonpollen aeroallergens and to food allergens. A mean of 1.5 allergens per patient was found in this group (Tables 2 and 4). Seven (14%) of them were sensitized to one allergen, and another 7 patients (14%) were sensitized to two allergens. The mean values of total serum IgE were 449.7 (±336.9) kU/L in the first group (patients with intermittent disease) and 934.2 (±765.8) kU/L in the second group (patients with persistent disease), presenting significant variability (Table 1). The rate of agreement between the results of the skin prick tests when performed and the results of RAST was high (Table 2). There was no consistent correlation between severity of allergic rhinitis and RAST classes, suggesting that probably antibody levels are only one of the factors that determine symptom severity [8].

Allergic rhinitis is a significant clinical problem in children. In the Mediterranean region there are characteristic climatic conditions, such as mildness of winter and poor rainfall during the summer, that facilitate the growing of a typical vegetation with production of allergenic pollen [1]. Rich and long pollinic seasons are, thus, favored, and the pollen grains of various plants can reach high atmospheric concentrations, causing severe clinical symptoms of rhinoconjunctivitis and asthma. The prevalence of allergic rhinitis in children in the Mediterranean countries has been reported to range from 9.4% to 16.8% [2, 9]. However, more than 40% of the children reported allergic rhinoconjunctivitis symptoms in the past [2, 10]. Most prevalent allergic plants with known clinical significance are grasses, *Parietaria* and *Olea europaea*.

*Parietaria* was the most important allergenic pollen in the children of our study. This is an *Urticacea* plant characteristic of Mediterranean flora, which has been found to be the most common cause of allergy in the Mediterranean countries, either in adults or in children [11]. D'Amato and Lobefalo [12] in a study conducted in Naples, found *Parietaria* as the most common allergen in adults, and Kontothanasi et al. [6], in a study of allergens in adult patients in western Athens, which is a neighbouring region to ours, reported this allergen as the second most common after *Graminae*, and especially *Dactilis glomerata*. Other species of *Urticaea* and *Cereales* were less commonly determined allergens in the subjects of our study.

From the trees, we found *Olea europeae* to be the most common aeroallergen, whereas *Cypressus sempervirens*, *Pinus pinea*, and *Populus alba* were less frequently identified. Olive trees are, only occasionally, found in the surroundings, but air currents carry their pollen from suburban areas. *Olea europeae* is a major tree producing allergenic pollen in the Mediterranean area [12, 13], and the same has been reported for cypress [14] and the other implicated trees [15].

In our study, most pollen aeroallergens were associated with intermittent rhinitis, whereas hypersensitivity in nonpollen allergens was associated with persistent rhinitis. We found house dust and mites (*Dermatophagoides pteronyssinus* and *Dermatophagoides farinae*) the most common allergens and this agrees to previous reports in Mediterranean countries. According to Verini et al. [13] a high incidence of positive reactions to *Dermatophagoides pt* and *fa* was found, exceeding 70%. Furthermore, Ramadan et al. [16] found a high incidence of mites in Lebanon. Lower rates were reported in other studies, as in the investigation by Erel et al. [17] in which an incidence of 20% was found in Turkey. Positivity to dog and cat allergens is of lower incidence, especially in countries where keeping house pets is not a common practice [16]. Food allergy as a potentially important factor in the pathogenesis of allergic rhinitis should, also, be noticed [18]. We found only two cases with allergy owed to egg and milk, but further investigation of a large number of children for food allergy is warranted.

Finally, we should mention that we found a mean of 2 allergens per patient in intermittent rhinitis and a mean of 1.5 allergens per patient in persistent rhinitis. Polysensitization has been also reported elsewhere, as in the study of Verini et al. [13], in which only 12% of children in a central Italian area were monosensitized, whereas the remaining were sensitized to 2-3 (56%) or even more allergens.

## 4. Conclusions

In conclusion, the detection of indoor and outdoor allergens in the region of Piraeus, based on skin prick tests and RAST tests, showed high incidence of grasses and food allergens, which is similar to other Mediterranean countries. Our results reflect the special characteristics of the region of Piraeus, which has a high population density and is polluted from industries, the port, and the heavy traffic. However, our subjects originated also from the country of the surrounding region, two nearby islands, and the rural area of Trizinia, resulting in the variety of allergens identified from our investigation.

## Figures and Tables

**Table 1:** Clinical and demographic features of our patients.

Patient	Sex	Age	Severity of rhinitis	Type of rhinitis	Other allergic symptoms	Total IgE level (kU/L)
(1)	M	11	Mild	I	−	224
(2)	M	7	Mild	I	−	785
(3)	M	10	Moderate/severe	I	+	454
(4)	F	14	Mild	I	+	134
(5)	F	10	Moderate/severe	I	+	224
(6)	F	11	Moderate/severe	I	+	900
(7)	M	6	Moderate/severe	I	−	342
(8)	F	12	Moderate/severe	I	+	345
(9)	F	13	Mild	I	−	296
(10)	M	6	Moderate/severe	I	+	199
(11)	F	13	Mild	I	+	400
(12)	M	11	Moderate/severe	I	+	556
(13)	F	10	Moderate/severe	I	+	256
(14)	F	9	Mild	I	+	292
(15)	F	11	Mild	I	−	378
(16)	M	8	Mild	I	+	444
(17)	M	7	Moderate/severe	I	+	1080
(18)	F	11	Moderate/severe	I	−	577
(19)	F	11	Mild	I	+	401
(20)	M	10	Moderate/severe	I	+	899
(21)	F	11	Moderate/severe	I	+	301
(22)	M	6	Mild	I	−	247
(23)	F	11	Mild	I	+	199
(24)	F	12	Moderate/severe	I	+	434
(25)	F	9	Moderate/severe	I	+	283
(26)	F	11	Moderate/severe	I	+	1903
(27)	M	11	Mild	I	−	390
(28)	M	13	Mild	I	+	122
(29)	F	10	Mild	I	−	345
(30)	M	13	Moderate/severe	I	+	256
(31)	F	13	Moderate/severe	I	−	412
(32)	M	11	Moderate/severe	I	−	796
(33)	F	11	Moderate/severe	I	+	156
(34)	M	12	Moderate/severe	I	+	414
(35)	F	12	Moderate/severe	I	+	342
(36)	F	12	Mild	I	+	404
(37)	M	11	Moderate/severe	P	+	1932
(38)	M	9	Mild	P	+	435
(39)	M	10	Moderate/severe	P	+	897
(40)	F	10	Moderate/severe	P	+	563
(41)	F	7	Mild	P	−	267
(42)	F	11	Mild	P	+	1789
(43)	M	14	Moderate/severe	P	+	567
(44)	F	13	Mild	P	−	278
(45)	M	10	Moderate/severe	P	+	387
(46)	F	13	Moderate/severe	P	+	1023
(47)	F	9	Moderate/severe	P	+	646
(48)	M	12	Moderate/severe	P	+	1009
(49)	F	13	Moderate/severe	P	+	2888
(50)	F	14	Moderate/severe	P	+	399

I: intermittent; P: perennial.

**Table 2:** Types of allergens, RAST classes, and skin reaction.

Patient	Sex	Age	Type of allergen	RAST class	Skin reaction
(1)	M	11	*Parietaria*	1	np
(2)	M	7	*Parietaria * *Pinus pinea*	2	np
(3)	M	10	*Parietaria* *Olea europea*	3	np
(4)	F	14	*Parietaria*	2	+
(5)	F	10	Grasses *Olea europea *	2	np
(6)	F	11	*Parietaria*	3	np
			Grasses *Pinus pinea *		
(7)	M	6	*Parietaria * *Olea europea*	2	np
(8)	F	12	*Parietaria * Grasses *Olea europea *	3	+ + −
(9)	F	13	*Parietaria*	1	+
(10)	M	6	Cereales *Olea europea * *Cypressus semp. *	2	np
(11)	F	13	*Parietaria * *Olea europea*	1	np
(12)	M	11	*Parietaria * Grasses	4	np
(13)	F	10	*Parietaria*	3	np
(14)	F	9	*Parietaria * *Urtica*	1	np
(15)	F	11	*Parietaria * *Pinus pinea * *Urtica*	3	np
(16)	M	8	*Parietaria * *Urtica*	1	np
(17)	M	7	*Olea europea*	4	np
(18)	F	11	*Parietaria * *Olea europea*	3	np
(19)	F	11	Cereales *Pinus pinea * *Populus alba *	1	np
(20)	M	10	*Parietaria* * Olea europea*	4	np
(21)	F	11	Grasses *Cypressus semp. *	1	np
(22)	M	6	*Parietaria* * Olea europea*	2	np
(23)	F	11	*Parietaria* * Urtica*	1	np
(24)	F	12	*Olea europea*	3	+
(25)	F	9	*Parietaria * Grasses *Olea europea *	2	np
(26)	F	11	*Parietaria * Grasses *Populus alba *	4	np
(27)	M	11	*Olea europea*	4	+
(28)	M	13	* Parietaria * *Pinus pinea*	1	− +
(29)	F	10	*Parietaria*	3	np
(30)	M	13	Grasses *Olea europea *	2	+ +
(31)	F	13	*Parietaria * Grasses *Urtica *	1	np
(32)	M	11	*Parietaria * *Populus alba*	3	np
(33)	F	11	*Parietaria * Cereales *Olea europea *	2	np
(34)	M	12	*Parietaria * *Populus alba*	1	np
(35)	F	12	*Parietaria*	3	np
			Grasses *Urtica *		
(36)	F	12	*Olea europea*	4	np
(37)	M	11	House dust Mites	2	np
(38)	M	9	House dust Mites	1	np
(39)	M	10	*Canis fam.*	2	np
(40)	F	10	*Canis fam. * *Parietaria*	4	np
(41)	F	7	*Felis dom.*	1	np
(42)	F	11	House dust Mites	2	np
(43)	M	14	Egg	3	+
(44)	F	13	House dust	2	+
(45)	M	10	Milk	1	np
(46)	F	13	Mites *Felis dom. *	3	+ +
(47)	F	9	*Canis fam.*	4	np
(48)	M	12	House dust Mites	2	np
(49)	F	13	House dust *Parietaria *	3	+ +
(50)	F	14	*Canis fam.*	4	+

np: not performed.

**Table 3:** Positive RAST (and skin prick test when performed) to various allergens in intermittent allergic rhinitis.

Allergens	Positive tests	
	*N*	%
Grasses	10	13.7
Cereales	3	4.1
*Parietaria*	27	36.9
*Urtica*	6	8.2
*Olea europea*	16	21.9
*Cypressus sempervirens*	2	2.8
*Pinus pinea*	5	6.9
*Populus alba*	4	5.5

Total	73	100

**Table 4:** Positive RAST (and skin prick test when performed) to various aeroallergens and food allergens in persistent allergic rhinitis.

Allergens	Positive tests	
	*N*	%
House dust	6	28.6
Mites (*D. pteronyssinus *and *D. farinae*)	5	23.8
Dogs (*Canis familiaris*)	4	19.0
Cats (*Felis domesticus*)	2	9.5
Egg	1	4.8
Milk	1	4.8
*Parietaria*	2	9.5

Total	21	100
